# Pelvico-Vesicostomy for Horseshoe Kidney With Severe Right Ureteric Stricture and Bilateral Hydronephrosis

**DOI:** 10.7759/cureus.32938

**Published:** 2022-12-25

**Authors:** Poosapati D Devasilpa Raju, Kiran Khedkar, Yashwant Lamture

**Affiliations:** 1 Surgery, Datta Meghe Institute of Medical Sciences, Wardha, IND; 2 Pediatric Surgery, Datta Meghe Institute of Medical Sciences, Wardha, IND

**Keywords:** cect, ureteric stricture, hydroureteronephrosis, pelvico vesicostomy, horse shoe kidney

## Abstract

Ureteropelvic junction (UPJ) obstruction is the most common renal anomaly observed in infants with congenital hydronephrosis. The present study presents a five-month-old infant with severe right ureteropelvic obstruction. Anderson-Hynes pyeloplasty intervention significantly improved renal function. The study concludes that early surgical intervention is the definitive treatment for avoiding kidney impairment and its complications.

## Introduction

Horseshoe kidney (HSK) is the most prevalent inborn renal fusion anomaly that affects 1 in 400-600 neonates [1-2]. The kidneys have a horseshoe-like structure, with renal tissue or a fibrous band at the lower poles connected and located between the superior mesenteric artery (SMA) and the aorta [3]. HSK is accompanied by concomitant abnormalities like urolithiasis, renal malignancies, multicystic dysplastic kidneys, pelvic ureteric junction obstruction, and hydronephrosis [4]. Recent years have seen an increase in the diagnosis of hydronephrosis in neonates and infants, typically brought on by stricture, posterior urethral valves, and obstruction of the ureteropelvic junction (UPJ) [5]. As a result, children are prone to renal failure and infections to take adequate and prompt surgical intervention [6].

Anderson-Hynes pyeloplasty is the gold-standard technique for UPJ obstruction [7]. However, only a few cases of open pyeloplasty were reported in infants younger than six months. Here, we report Anderson-Hynes open pyeloplasty performed on a five-month-old infant diagnosed with HSK and right ureteropelvic obstruction due to a right ureteric stricture.

## Case presentation

**Figure 1:**
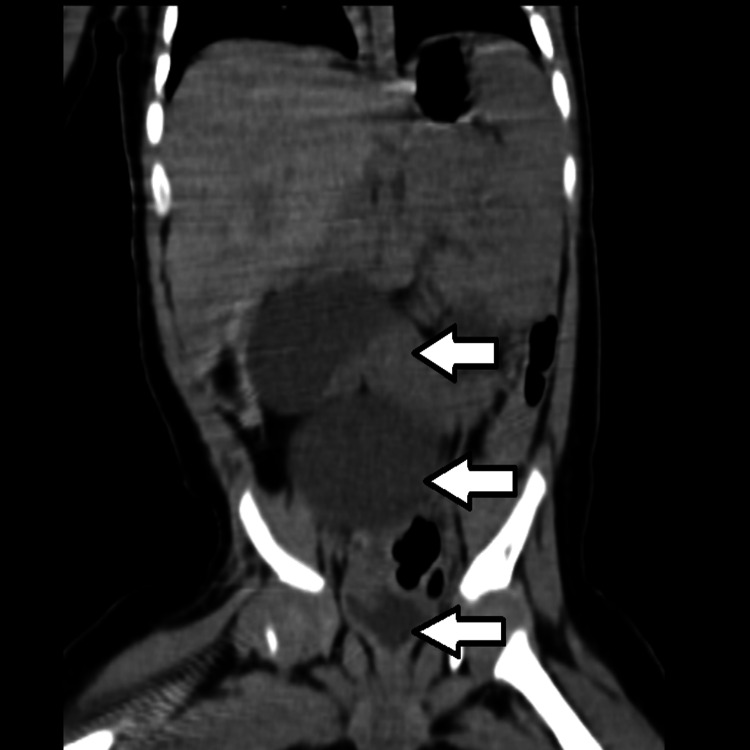
Coronal section of CT urography. Arrows show a grossly dilated renal pelvis with a severe right ureteric stricture and the bladder in a continuous sequence.

**Figure 2:**
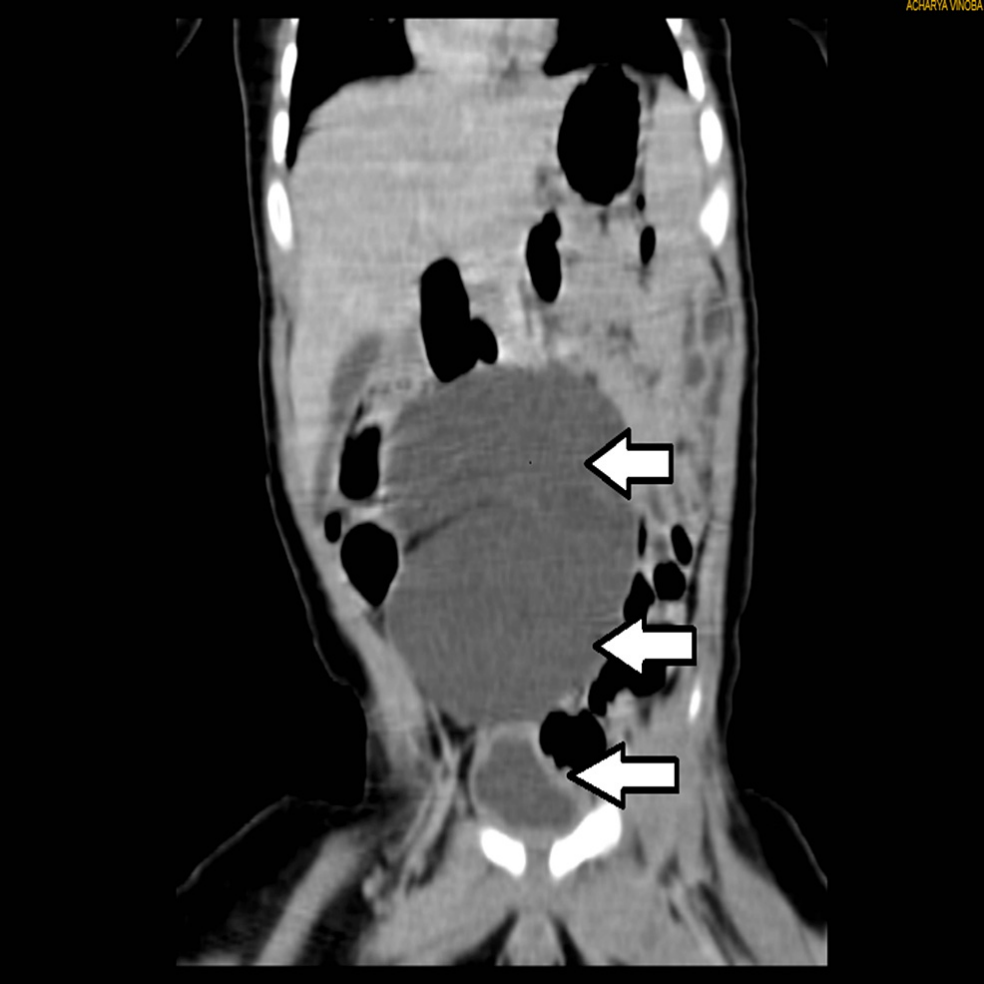
CT urography coronal cut. From top to bottom, the topmost and the middle arrows show a massively dilated renal pelvis with the ureter hardly visualized. The arrow over the bottom shows the bladder in close communication with the renal pelvis.

**Figure 3:**
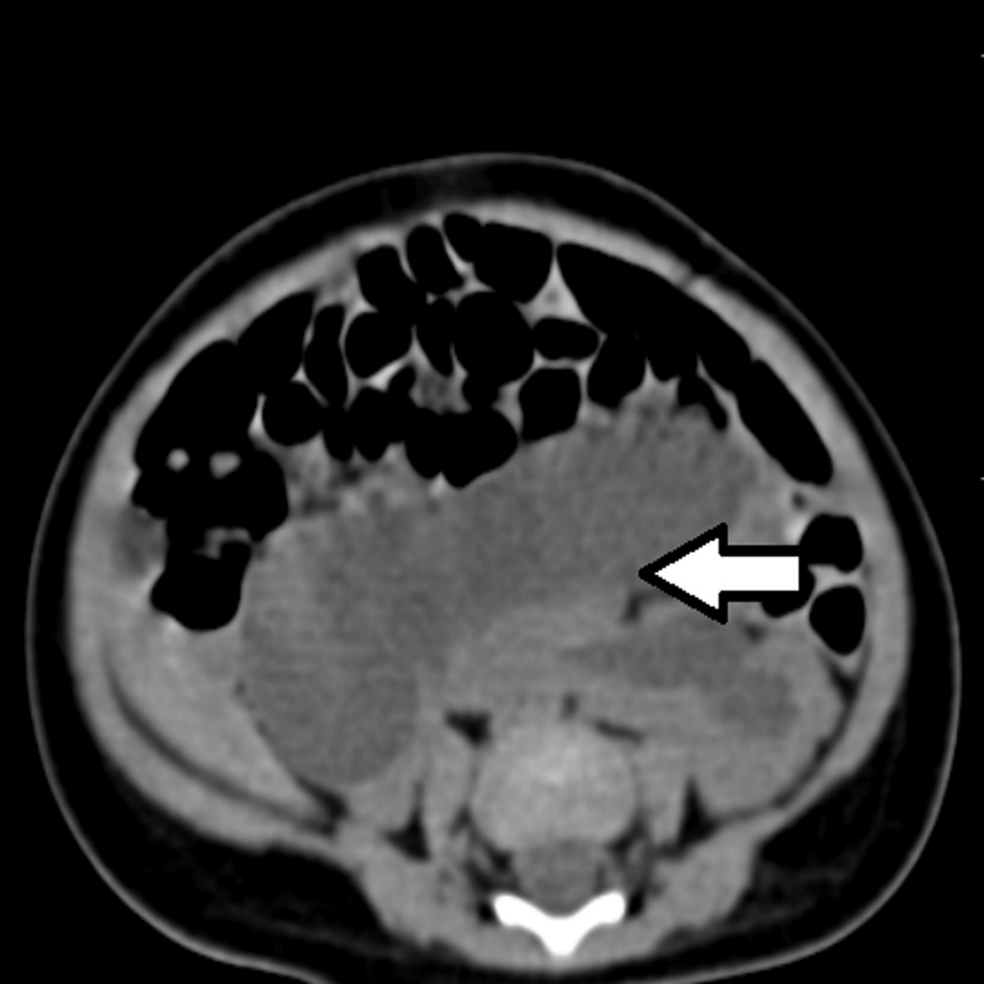
CT urography axial cut showing a horse-shoe shaped kidney occupying the retroperitoneum.

A five-month-old male infant with a known case of HSK since birth was admitted to the hospital for CT urography. On CT urography, the patient was found with severe bilateral hydroureteronephrosis, right pelvic ureteric junction obstruction (Figures 1-3), and partial obstruction of the left ureter, with an HSK. Complete blood analysis and all routines were done, followed by an Anderson-Hynes pyeloplasty performed on the patient under general anesthesia. Pre-operatively, the patient had the following significant labs: Hemoglobin (Hb) 14 gm, WBC 12,000 cells/cumm, and platelet 2,60,200 cells/cumm. Pre-operative kidney function test (KFT) was creatinine 1.8, urea 32, sodium 148, and potassium 4.0.

**Figure 4:**
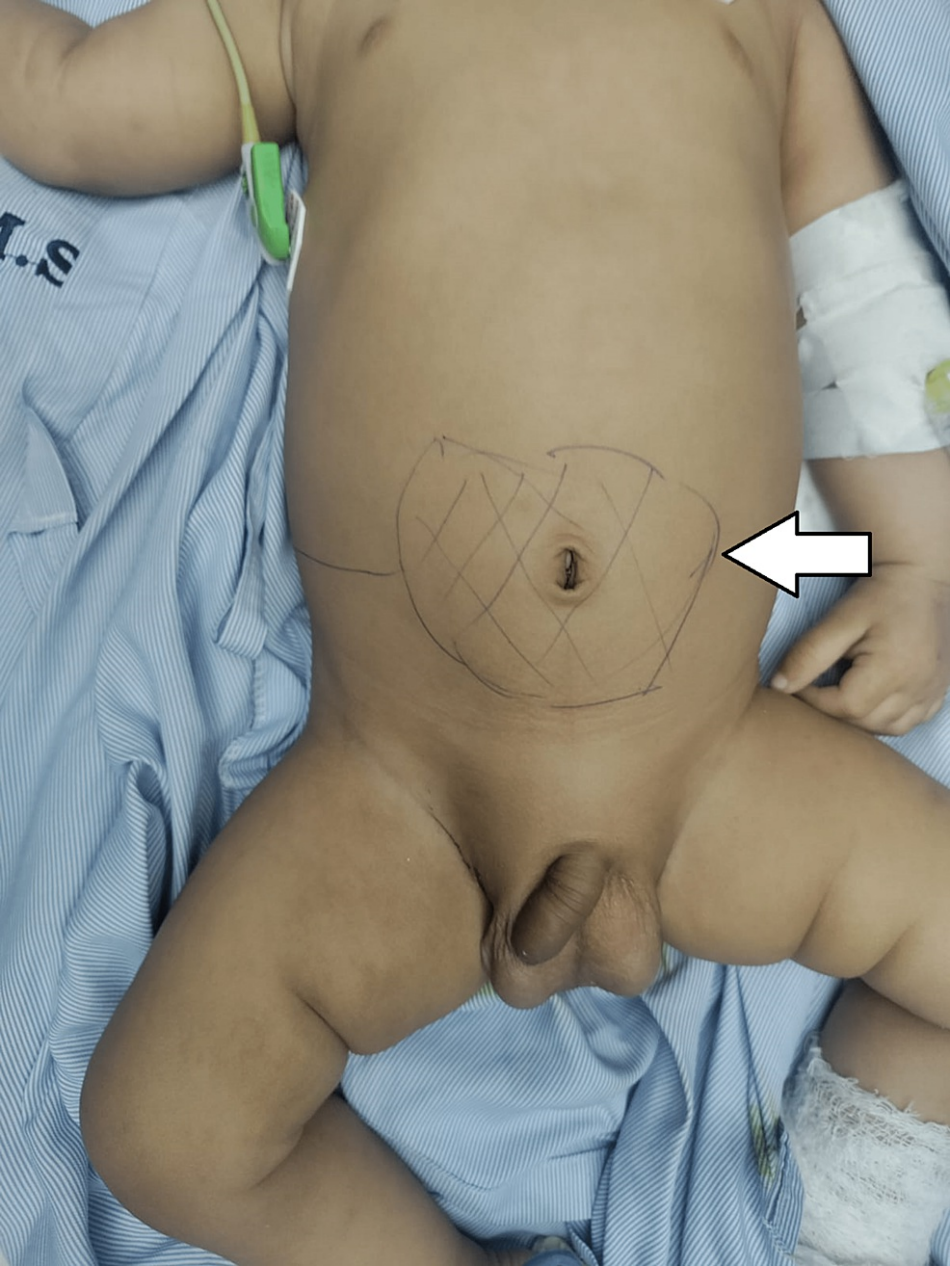
Anatomical marking of the grossly dilated renal pelvis below the horseshoe-shaped kidney.

**Figure 5:**
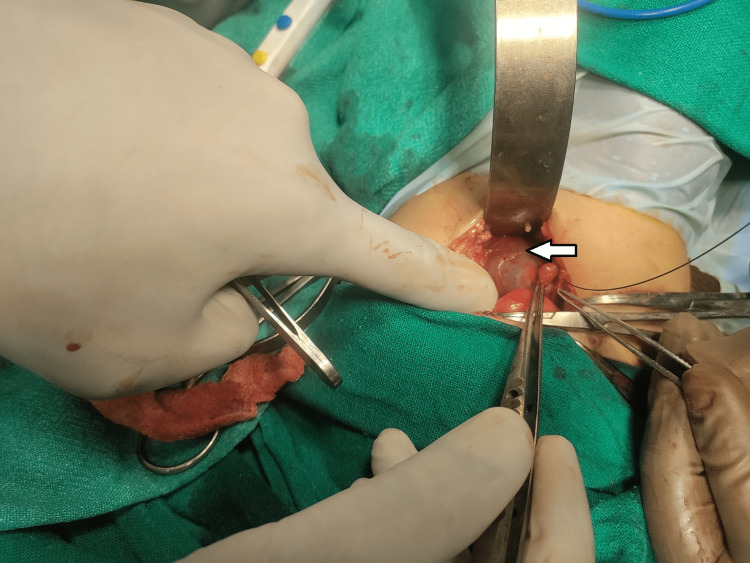
After making the incision and carefully dissecting up to the retroperitoneum, a grossly dilated right renal pelvis with a right ureteric stricture was identified.

**Figure 6:**
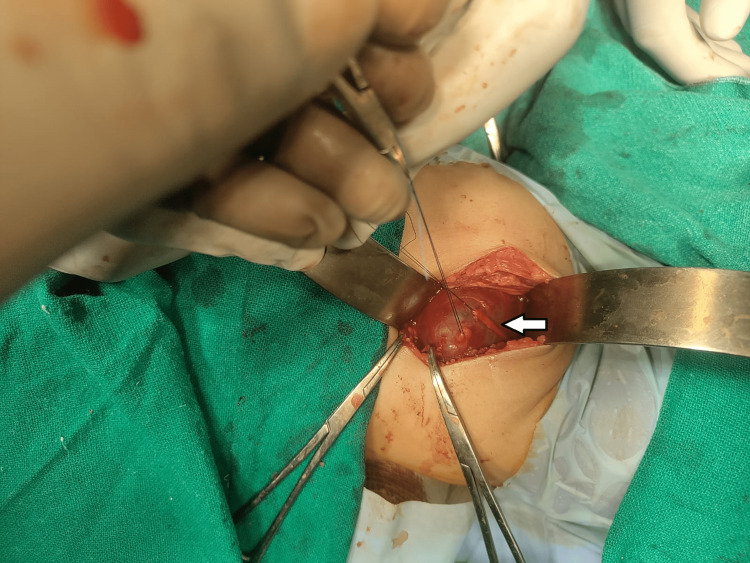
The entire right ureteric stricture was excised, and an end-to-end anastomosis was done where the dilated right renal pelvis was sutured with the bladder after placing a 3/12 DJ stent.

A rough anatomical landmark of the dilated renal pelvis was marked over the abdomen surrounding the umbilicus prior to the incision (Figure 4). The incision line was drawn just lateral to the anatomical landmark of the dilated renal pelvis extending to the right lumbar region. The incision was taken over the landmark and deepened until the peritoneum was opened. The small bowel and large bowel were lateralized after careful dissection. Once the retroperitoneal cavity was reached, a grossly dilated right renal pelvis with a stenosed ureter along the right side extending from the right renal pelvis to the ureterovesical junction was identified (Figure 5). End-to-end vesicopelvic anastomosis was performed over a 3/12 sized DJ stent in situ, and a number 20 size abdominal drain was inserted and fixed (Figure 6).

**Figure 7:**
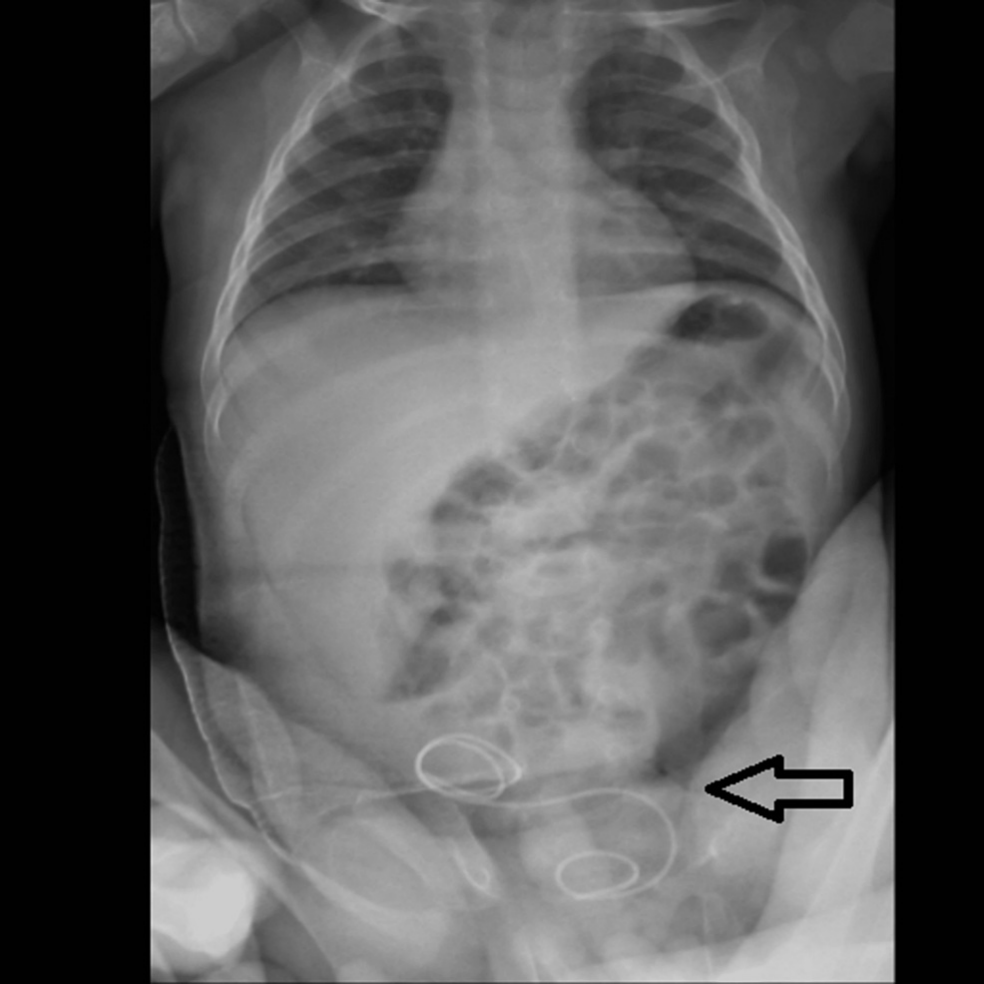
X-ray of the erect abdomen showing a 3/12 DJ stent in situ postoperatively.

Following surgery, the patient was admitted to the pediatric ICU, where the patient was managed conservatively. The patient had no episodes of fever or vomiting. After discharge from the pediatric ICU, the patient was shifted to the general ward. A post-operative X-ray erect abdomen was done to look for the DJ stent in situ (Figure 7).

**Figure 8:**
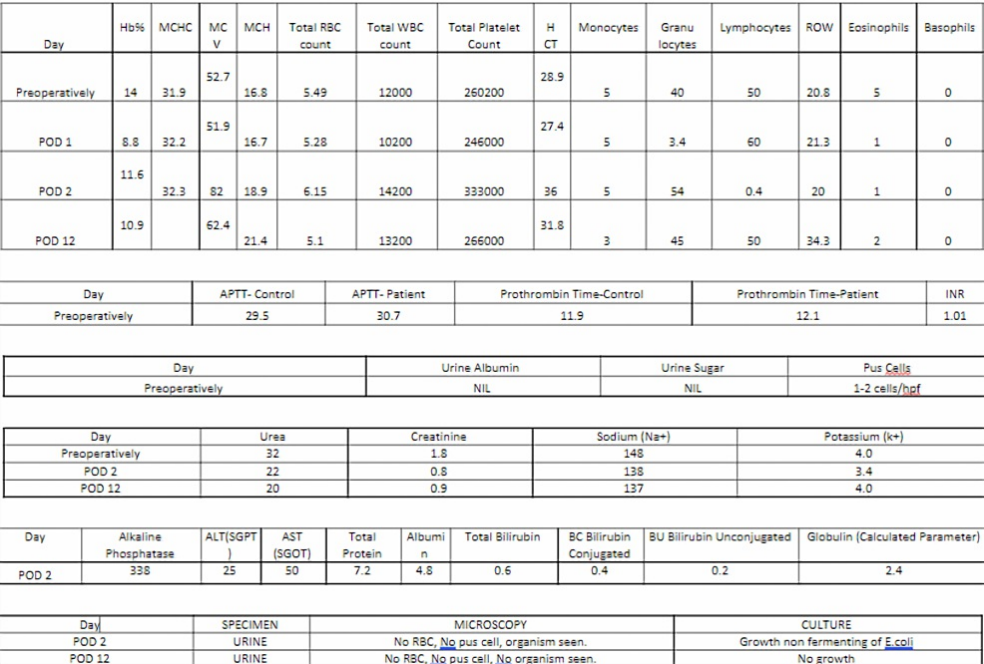
Investigations of the same patient. The image shows a comparative improvement in the KFT from the preoperative to post-operative period. KFT: Kidney function test.

The patient was conscious, active, vitally, and hemodynamically stable at discharge. The drain was removed under aseptic precautions at POD 8. Complete suture removal was done by POD 12 and was scheduled for follow-up visits to the urology outpatient department at regular intervals. Postoperatively, there was a significant improvement in renal function. The KFT values were as follows: POD 2 - creatinine 0.8, urea 22, sodium 138, and potassium 3.4; POD 12 - creatinine 0.9, urea 20, sodium 136, and potassium 4.0, as mentioned in Figure 8.

## Discussion

The most prevalent cause of juvenile hydronephrosis is UPJ obstruction, which affects 1 in 1000-2000 newborns [8]. Among them, at least 1/3rd of individuals had ureteropelvic obstruction as well. Therefore, early detection and treatment of obstructive uropathy in children are critical to preserving functioning nephrons, mainly in infants and neonates, where immature nephrons are more vulnerable to pressure. In the present study, a five-month-old male infant identified with a grossly dilated right renal pelvis with a stenosed ureter was rectified surgically. Postoperatively, urine output was monitored regularly. The 24-hour urine routine and microscopy were found to be normal. The patient was discharged with a healthy scar line.

Early surgical intervention has drawn criticism for subjecting patients to the dangers of unneeded surgery. However, rapid surgical correction was required in infants to preserve renal function and correct urine flow. A multicenter prospective randomized trial was conducted in 1998 by the Society for Fetal Urology to assess and compare the natural course of UPJ treated with surgery and without surgery. Thirty-two newborns with grade 3 or 4 hydronephrosis and less than 40% relative renal function were randomized into immediate pyeloplasty and observation groups. The results showed that renal function had stabilized in both groups, but the surgical group had quickly improved hydronephrosis and urine drainage. Also, 25% of the patients in the observation group had switched to pyeloplasty [9].

Similarly, Chertin B et al. reported that open Anderson-Hynes dismembered pyeloplasty was performed on two groups of patients: Group 1, with a mean age of 11 months with prenatal hydronephrosis, and group 2, with a mean age of five years with neonatal hydronephrosis, who were lost to follow-up and presented with symptoms consistent with UPJ obstruction. After more than three years of follow-up, Group 2's poor relative renal function (30%) was considerably worse than Group 1's, although both groups' hydronephrosis had greatly improved. The study concluded that those with early-stage UPJ blockage should be closely and thoroughly monitored [10].

In the present study, the patient was followed up for six months to evaluate any postoperative complications. According to many authors, major complication following pyeloplasty surgery, such as anastomosis leak, happens within a year of the procedure. Postoperative follow-up duration significantly affects the incidence of problems [7] [11]. The minimal observation period in articles describing the control following pyeloplasty surgery was six months and twelve months [12-13]. In this study, no major postoperative complications were observed during six months of follow-up. The patient's suture was completely healed. The patient was active and healthy, with normal urine output and serum creatinine levels.

## Conclusions

This case highlights the need for early pyeloplasty intervention with infants detected with severe uteropelvic blockage. It also highlights the necessity of prenatal screening for the early detection and treatment of UPJ blockage. Pyeloplasty leads to prompt improvements in renal function, improvements in ultrasonographic images, and symptom remission. However, appropriate interval postoperative follow-up is necessary to avoid recurrence and prevent irreversible renal function failure.
